# The efficacy of a nitric oxide-releasing formulation on nares isolated Methicillin-Resistant *Staphylococcus aureus* in porcine wound infection model

**DOI:** 10.3389/fcimb.2024.1501360

**Published:** 2024-12-03

**Authors:** Stephen C. Davis, Joel Gil, Michael Solis, Ryan Strong

**Affiliations:** Dr. Phillip Frost Department of Dermatology & Cutaneous Surgery, University of Miami Miller School of Medicine, Miami, FL, United States

**Keywords:** nitric oxide, intranasal gel, biofilm, nasal infection, Methicillin-Resistant *Staphylococcus aureus*, porcine wound model

## Abstract

**Background:**

The colonization of *Staphylococcus aureus* (SA) acquired in nosocomial infections may develop acute and chronic infections such as Methicillin-Resistant *Staphylococcus aureus* (MRSA) in the nose. As a commensal microorganism with the ability to form a biofilm, SA can dwell on the skin, nostrils, throat, perineum, and axillae of healthy humans. Nitric oxide (NO) is an unstable gas with various molecular functions and has antimicrobial properties which are converted into many potential treatments.

**Methods:**

Methicillin-Resistant *Staphylococcus aureus* MRSA BAA1686 isolated from nasal infection was used in a porcine wound infection model. Deep partial-thickness wounds (10mm x 7mm x 0.5mm) were made on three animals using a specialized electrokeratome. All wounds were inoculated and then covered with polyurethane film dressings for biofilm formation. After 48 hours, three wounds were recovered from each animal for baseline enumeration. The remaining wounds were randomly assigned to six treatment groups and treated once daily. The treatment groups are as follows: NO topical ointments concentrations of 0.3, 0.9 and 1.8%, Vehicle Ointment, Mupirocin 2%, and Untreated Control. Microbiological recoveries were conducted on day 4 and day 7.

**Results:**

The greatest efficacy observed from the NO formulations against MRSA BAA1686 was the 1.8% concentration. This agent was able to reduce more than 99% of bacterial counts when compared to Baseline, Vehicle Ointment, and Untreated Control wounds on both assessment days. Mupirocin 2% was the overall best treatment against MRSA BAA1686 on both assessment days, with a significant reduction (p ≤ 0.05) of 4.70 ± 0.13 Log CFU/mL from day 4 to day 7.

**Conclusions:**

Overall, the positive control Mupirocin 2% was the most effective in eliminating MRSA BAA1686 throughout the study. This experiment demonstrated a downward trend from the highest concentration of NO topical ointment formulations to the lowest concentrations on both assessment days (0.3% - 1.8%). Out of all NO topical ointments, the highest concentration (1.8%) was the most effective with the potential to be an alternative treatment against a MRSA nasal strain biofilm.

## Introduction

Many skin and soft tissue infections (SSTIs) are commonly caused by *Staphylococcus aureus* (SA), a gram-positive pathogen that colonizes anterior nares, and are further complicated by the prevalence of methicillin-resistant *S. aureus* (MRSA) infections correlated to nasal carriage strains (Costa et al., 2024). These pathogens can easily transfer to various body parts, and even spread to other individuals in infected environments (Dexter et al., 2024).

The identification of MRSA colonization to prevent these infections remains a considerable challenge with an associated economic burden stemming from screenings for pathogens, materials for prevention, surveillance and isolation of patients (McKinnell et al., 2015). The nasal passage is vulnerable as particles can easily enter the respiratory tract while it is performing its common functions such as humidifying air, trapping and removing dust particles, and draining paranasal sinuses and lacrimal ducts (Wos-Oxley et al., 2016). Cases of compulsive nose picking (rhinotillexomania) have shown elevated risks to epistaxis and nasal infections by SA, stemming from damage to the interior nares from constant penetration (Gupta and Dhingra, 2018).

The human nose is a biological reservoir for SA strains with MRSA being one of the leading causes of bacterial infections and a risk factor for nosocomial infections within hospital settings and developing countries (Wolde et al., 2023). The spread of SA from nares to surgical sites can transfer through either direct contact, indirect contact from contaminated instruments, airborne within an operating room or hematogenous transmission (Troeman and Kluytmans, 2023). One study demonstrated that nasal swabs of atopic dermatitis patients found various isolates such as MRSA, methicillin-susceptible SA (MSSA), methicillin-resistant *Staphylococcus epidermidis* (MRSE), and methicillin-susceptible *Staphylococcus epidermidis* (MSSE) (Augusto de Oliveira et al., 2024).

Nitric oxide (NO) is an endogenously produced small gas molecule with many functions throughout the body and that has bactericidal properties (Cartwright et al., 2022). Literature has demonstrated the benefits of NO throughout the body and the phases it goes through, with notable interactions related to healing and terminating pathogens (Belenichev et al., 2024). Due to its short lifespan, NO has been incorporated into various delivery systems such as hydrogels, ointments, and nanoparticles to control the storage and release rate of NO (Choi et al., 2020). In today’s market, NO is found in ventilation devices, nasal sprays, solutions, topical agents, dressings and bandages due to its multiple benefits (Bryan, 2015; Gonzalez et al., 2023). With its incorporation into multiple antibacterial agents, NO has shown to be an effective antimicrobial against gram positive and negative bacterial strains, capable of disrupting the formation of biofilms (Cui et al., 2024).

Biofilms are colonies of bacteria cells that are surrounded by an extracellular polymeric substance (EPS) matrix that provides structural support and resistance, along with elastic properties that restores its form after deforming (Almatroudi, 2024). Biofilms increase resistance to antimicrobials and the host immune response which leads to frequent incidences of bacteremia and sepsis in hospital settings (Gulati et al., 2024). Studies have shown that the nasal carriage colonization of SA strains develops even when unexposed in a hospital setting and are more susceptible if exposed (Sharma et al., 2020; Yang et al., 2022).

The efficacious of NO in *in-vitro* studies have shown success against various microbes such as: MSSA, MRSA, *Streptococcus pyogenes*, *Enterococcus faecalis*, *Klebsiella pneumoniae*, *Escherichia coli* (*E. coli*), *Pseudomonas aeruginosa* (PA), *Acinetobacter baumannii* (AB) and even a fungi like *Candida albicans* (CA) (Abdel Azim et al., 2024). Ghaffari et al. has supported findings of other studies that against these bacterial pathogens, NO as topical antimicrobial agent is effective (Ghaffari et al., 2006). The antimicrobial effect of NO demonstrated efficacy in viruses, bacteria, fungi, and parasites, while in animal models enhanced the abilities of host to fight infectious agents and reduced microbial proliferation (Jones et al., 2010). This study measures the efficacy of NO topical ointments of various concentrations, on MRSA BAA1686 biofilms clinically isolated from nares in a deep partial thickness porcine wound infection model. The ointment formulation evaluated in this study, NVN4428, has previously demonstrated antimicrobial efficacy *in vitro* against a multitude of *S. aureus* strains (Carbó and Croasdell, 2013). A porcine model is relevant since the skin morphology and immunome of swine are analogous to humans with these similarities yielding valuable findings on *in vivo* studies of wound healing and infections (Sullivan et al., 2001; Goodwine et al., 2019). This study found that topical application of nitric oxide ointment effectively reduced pathogenic burden in wounds infected with a nasal MRSA isolate.

## Materials and methods

### Experimental animals

The protocol for this study was reviewed and approved by the University of Miami’s Institutional Animal Care and Use Committee. Three young female specific-pathogen-free Yorkshire pigs, 2-3 months old, weighing 35-40 kg were used for this study.

### Animal preparation

Each of the animals were sedated for all procedures and given analgesics throughout the study. The back and sides of each animal were clipped with standard animal clippers, washed with non-antibiotic soap and sterile water, and then blotted dry with sterile gauze.

### Wounding

**Figure 1:**
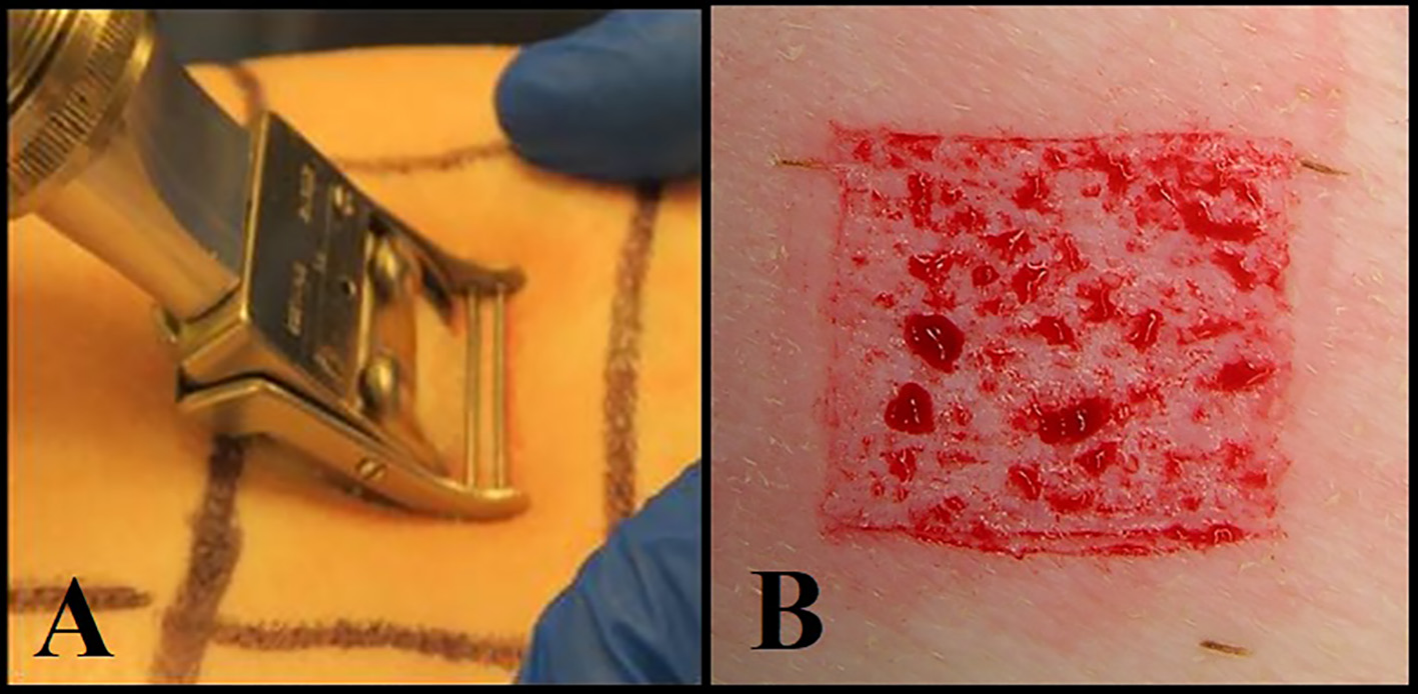
**(A)** Using an electrokeratome to create the wounds. **(B)** An example of a deep partial thickness wound.

**Figure 2:**
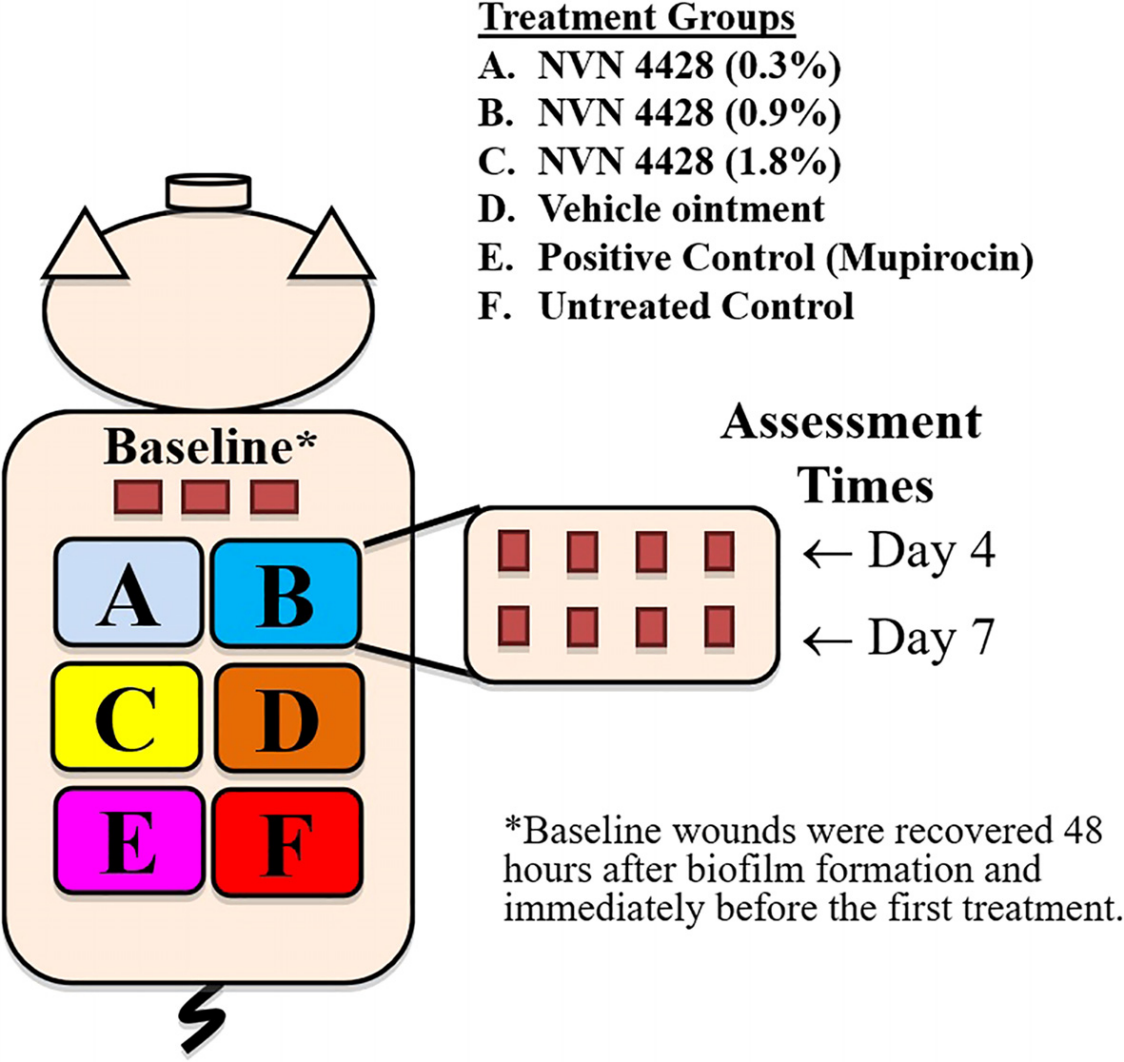
Experimental design for 3 animals infected with Methicillin-Resistant *Staphylococcus aureus* MRSA BAA1686.

Deep partial-thickness wounds (10 mm x 7 mm x 0.5 mm) were created on the paravertebral and thoracic areas of each animal using a specialized electrokeratome device fitted with a 7 mm blade (**Figure 1**) (Davis et al., 2015; Davis et al., 2017; Davis et al., 2019). Fifty-one (51) deep partial-thickness wounds were created on each of the animals resulting in a total of 153 wounds. Each wound was separated from one another by 5 - 7 cm of unwounded skin, and randomly assigned to one of six treatment groups. All treatment groups consisted of eight wounds per animal, and three wounds per animal were designated for baseline recovery (**Figure 2**).

### Inoculation

A fresh culture of MRSA which was clinically isolated from nasal infections and deposited in the American Type Culture Collection (ATCC) BAA1686 (MRSA BAA1686) was used in this study. Serial dilutions were performed from a scraping of overnight growth in saline to create an inoculum suspension with a concentration of 10^6^ CFU/mL, which was then quantified by plating serial dilutions of the suspension onto specific media. The inoculum suspension was vortexed and twenty-five (25) (Davis et al., 2019) µL aliquots of the suspension were deposited into the center of each wound for all animals. After inoculation, wounds were covered individually for 48 hours with a polyurethane film dressing (Tegaderm; 3M, St. Paul, MN) to allow for biofilm formation. We have previously shown mature biofilm formation in wounds 48 hours after inoculation using a scanning electron micrograph (Davis et al., 2008). The dressings were secured in place with surgical tape and self-adherent bandages (Coban, 3M).

### Treatment regimen

**Figure 3:**
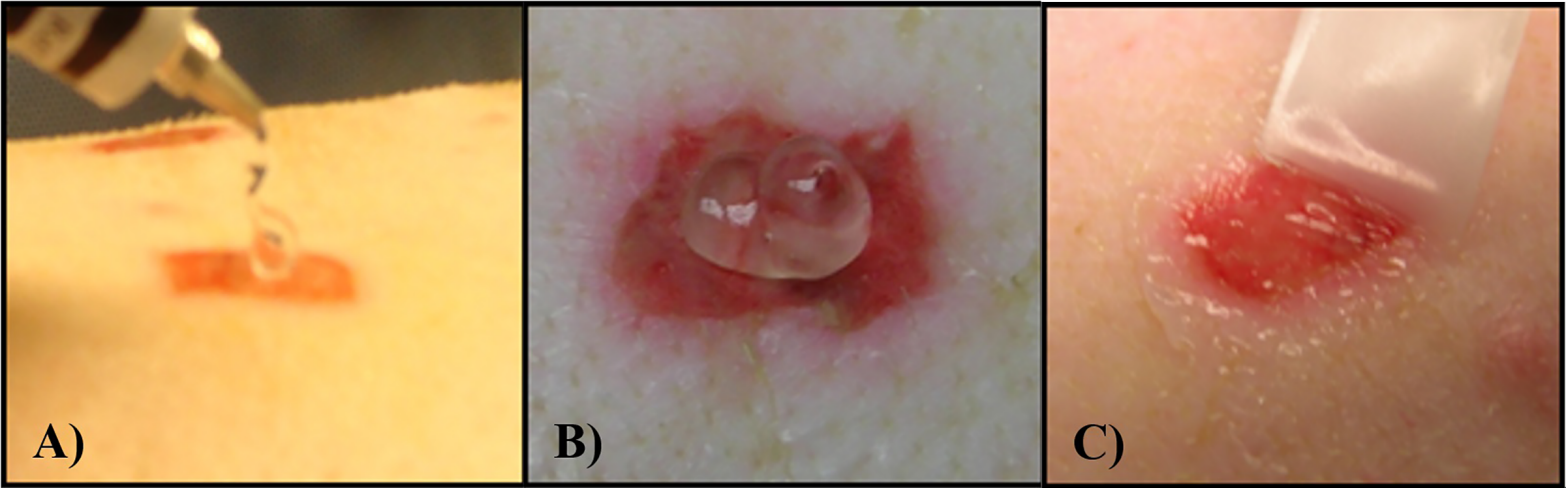
**(A)** Example of formulation being applied to the wound. **(B)** Example of 200 mg of formulation on deep partial thickness wound. **(C)** Formulation being spread around the wound with a sterile Teflon spatula before covering with a Tegaderm dressing.

The polyurethane film dressings were removed 48 hours after inoculation. Three wounds per animal were recovered for baseline bacterial counts. The remaining wounds were randomly assigned to six treatment groups, all treatment groups consisted of eight wounds per animal (**Figure 2**). A proprietary ointment (NVN 4428) was used at various concentrations for these studies (Doxey et al., 2020). The treatment groups were as follows: NVN 4428 (0.3%), NVN 4428 (0.9%), NVN 4428 (1.8%), Vehicle Ointment, Positive Control (Mupirocin) and Untreated Control. NO formulations were prepared immediately prior to application. Each wound received 200mg of assigned formulations that were applied daily after 48 hours of inoculation and the dressings were replaced after each treatment application (**Figure 3**). After treatment application, wounds from each treatment group were covered with a polyurethane film dressing to prevent cross contamination.

### Microbiology assessment

Three wounds per animal were recovered 48 hours after inoculation to obtain the baseline bacterial counts. Four wounds from each treatment group per animal were recovered on days 4 and 7. Each wound was recovered for microbiology assessment only once. Recovery was conducted by placing a sterile surgical steel cylinder (22 mm diameter) at the center of the wound. One (1) mL of a scrub solution was dispensed via pipette into the cylinder. The site was then scrubbed with a sterile Teflon spatula for thirty (Pastar et al., 2013) seconds. Serial dilutions were performed for all recovery samples and the Spiral Plater System (Spiral Biotech, Norwood, MA) was used to quantify the bacterial load. The Spiral Plater System deposits a 50 μL aliquot of the scrub suspension over the surface of a rotating agar plate. Oxacillin Resistance Screening Agar Base (ORSAB) was used to quantify the bacterial count of MRSA. The selective media plates were incubated aerobically at 37°C for 24 - 48 hours, then the number of viable colonies was counted, and the Log CFU/mL was calculated (Pastar et al., 2013).

## Results

**Figure 4:**
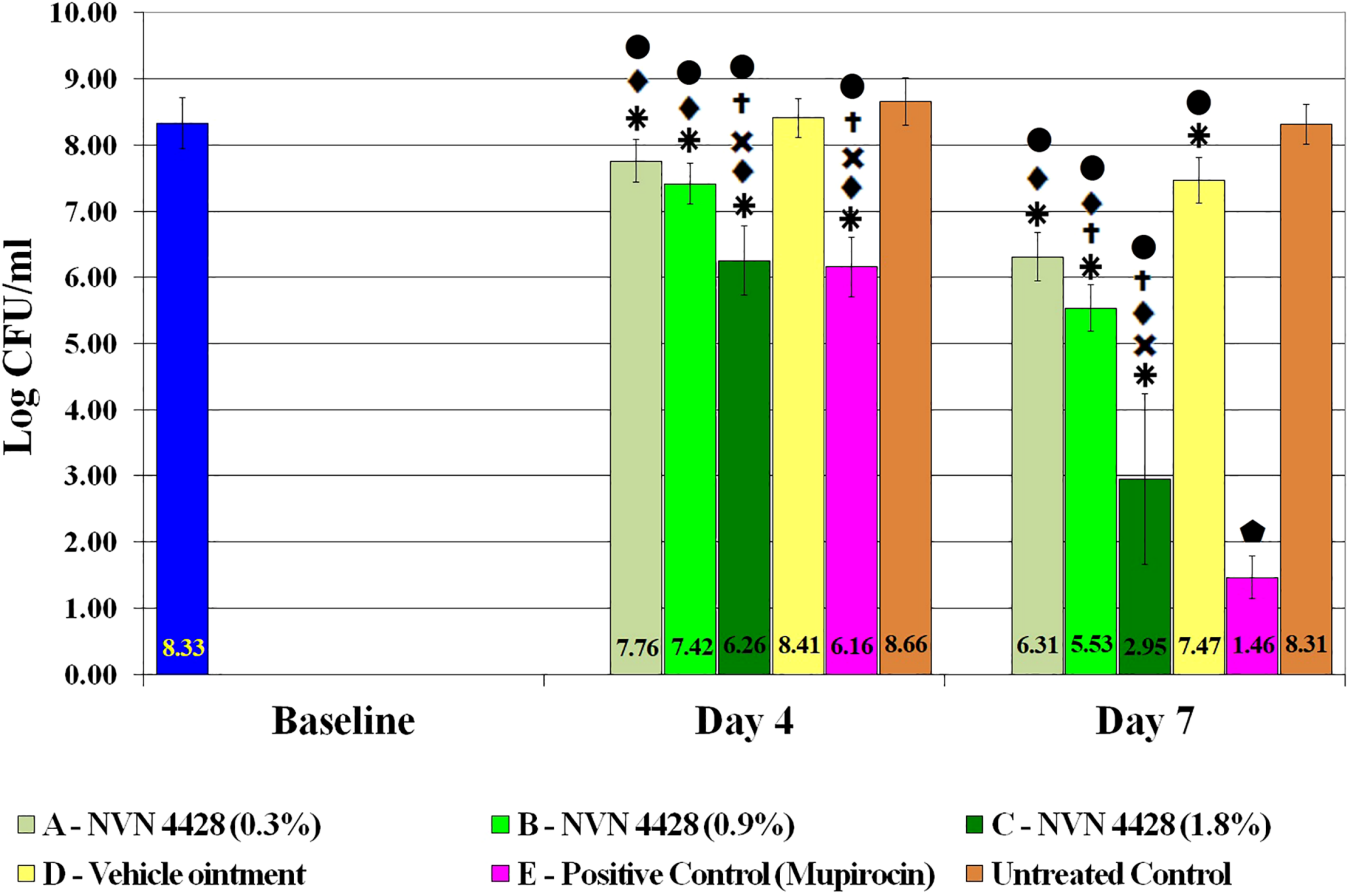
Effects of treatment groups on bacterial counts of MRSA BAA1686 infections between treatments. ^
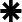
^p ≤ 0.05 compared to Baseline. ^
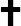
^p ≤ 0.05 compared to NVN 4428 (0.3%). ^
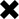
^p ≤ 0.05 compared to NVN 4428 (0.9%). ^
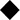
^p ≤ 0.05 compared to Vehicle Ointment. ^
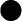
^p ≤ 0.05 compared to Untreated Control. ^
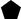
^p ≤ 0.05 compared to all treatment groups.

The baseline wounds after a 48-hour biofilm formation and prior to treatment application were recovered and quantified to determine the effect of the NO formulations. The observed baseline bacterial burden was 8.33 ± 0.39 Log CFU/mL of MRSA BAA1686 (**Figure 4**). Both Mupirocin 2% (Positive Control) and NVN 4428 (1.8%) treatment groups had the most significant MRSA BAA1686 reductions (p ≤ 0.05) of 99.32% and 99.15% (2.17 ± 0.06 and 2.07 ± 0.14 Log CFU/mL, respectively) when compared to the baseline wounds on day 4. Similarly, when compared to Untreated Control, Mupirocin 2% and NVN 4428 (1.8%) resulted in significant (p ≤ 0.05) reductions of MRSA on day 4 with reductions of 99.69% (2.50 ± 0.09 Log CFU/mL) and 99.61% (2.41 ± 0.17 Log CFU/mL) observed, respectively. Compared to the Untreated Control wounds, NVN 4428 (0.9%) treatment significantly (p ≤ 0.05) reduced 1.25 ± 0.05 Log CFU/mL (94.33% reduction) of bacteria on day 4 (**Figure 4**). Furthermore, the NVN 4428 (0.3%) treatment significantly (p ≤ 0.05) reduced MRSA BAA1686 by 0.90 ± 0.03 Log CFU/mL (87.51%) when compared to the Untreated Control wounds on day 4. Lastly, when compared to Vehicle Ointment on day 4, NVN 4428 (1.8%) significantly (p ≤ 0.05) reduced the bacterial count by 2.16 ± 0.23 Log CFU/mL or 99.30%. The NVN 4428 (0.9%) and NVN 4428 (0.3%) also resulted in significant (p ≤ 0.05) bacterial reductions of 89.86% and 77.66%, respectively, on day 4 compared to Vehicle Ointment.

By Day 7, both Mupirocin 2% and NVN 4428 (1.8%) treatment groups continued to exhibit the lowest amounts of MRSA BAA1686 with bacterial counts of 1.46 ± 0.32 Log CFU/mL and 2.95 ± 1.29 Log CFU/mL, respectively (**Figure 4**). These treatments resulted in significant (p ≤ 0.05) bacterial reductions of 99.99% when compared to the baseline wounds containing reductions of 6.87 ± 0.06 Log CFU/mL and 5.38 ± 0.91 Log CFU/mL, respectively. Similarly significant (p ≤ 0.05) MRSA reductions of 99.99% were confirmed on day 7 when comparing Mupirocin 2% and NVN 4428 (1.8%) treated wounds to the Untreated Control wounds.

The NVN 4428 (0.9%) treatment group on day 7 significantly (p ≤ 0.05) reduced the counts of bacteria by 2.79 ± 0.03 Log CFU/mL (99.84%) compared to the baseline wounds and 2.78 ± 0.05 Log CFU/mL (99.83% of reduction) when compared to Untreated Control wounds. The wounds treated with NVN 4428 (0.3%) exhibited significant (p ≤ 0.05) bacterial reductions of 99.03% (2.01 ± 0.03 Log CFU/mL) when compared to the baseline wounds and 99.00% (2.00 ± 0.05 Log CFU/mL) when compared to Untreated Control wounds.

On day 7, the NVN 4428 (1.8%), NVN 4428 (0.9%), and NVN 4428 (0.3%) treatment groups each significantly (p ≤ 0.05) reduced bacterial burden compared to Vehicle Ointment, with respective reductions of 4.52 ± 0.94 Log CFU/mL (99.99%), 1.94 ± 0.00 Log CFU/mL (98.84%) and 1.16 ± 0.01 Log CFU/mL (93.01%). When comparing Vehicle Ointment to the baseline wounds on Day 7, there was a significant (p ≤ 0.05) difference of 0.86 ± 0.04 Log CFU/mL with a bacterial reduction of 86.11%. Additionally, a significant (p ≤ 0.05) reduction of 0.85 ± 0.04 Log CFU/mL (85.74%) of MRSA was observed when comparing the Vehicle Ointment treatment to Untreated Control wounds. There was no significant difference detected between baseline and Untreated Control wounds on both assessment days.

**Figure 5:**
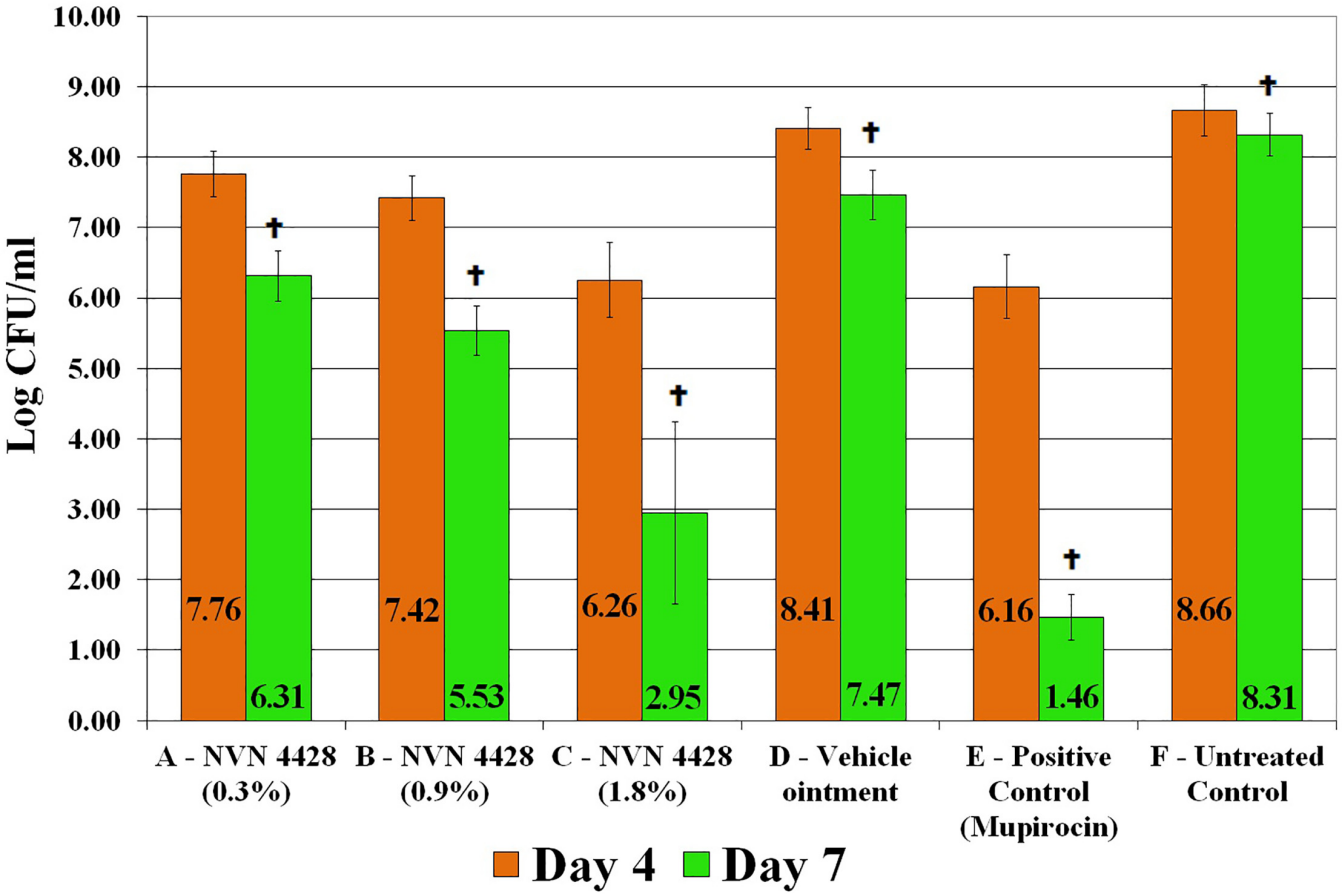
MRSA BAA1686 bacterial counts for each treatment group between assessment days.

There was significance observed in the reduction of MRSA BAA1686 amounts among all treatment groups between each assessment day (**Figure 5**). The most significant (p ≤ 0.05) bacterial reductions were observed from treatment groups Mupirocin 2% and NVN 4428 (1.8%) with bacterial reductions of 4.70 ± 0.13 Log CFU/mL (99.99%) and 3.31 ± 0.76 Log CFU/mL (99.95%), respectively, between assessment day 4 and day 7. The remaining NO treatment groups, both NVN 4428 (0.9%) and NVN 4428 (0.3%) + Hydrogel, each demonstrated significant (p ≤ 0.05) reductions of MRSA between both assessment days with differences of 1.88 ± 0.04 Log CFU/mL and 1.45 ± 0.04 Log CFU/mL (98.69% and 96.42%), respectively. The Vehicle Ointment + Hydrogel and Untreated Control treatments from day 4 to day 7 exhibited significant (p ≤ 0.05) but comparatively minor differences of <1 Log CFU/mL in MRSA BAA1686 burden between the assessment days during the study.

## Discussion

The nose is a protective barrier with epithelial cells able to create an immune response, but *S. aureus* is a persistent pathogen with an invasive infectious potential that interacts with multiple host and bacterial factors (Mulcahy and McLoughlin, 2016). Further, MRSA is a common colonizer within nares in health care settings and nasal screenings are frequently implemented for infection control and prevention purposes for vulnerable inpatients and outpatients (Coye et al., 2023). A report from the National Nosocomial Infections Surveillance stated that MRSA in intensive care units (ICUs) has, on average, accounted for 57% of *S. aureus* isolates causing nosocomial colonization or infections per data ranging from 1992 to 2002 (NNIS System, 2003). According to Davis, Kepler A et al., there’s an increased risk of MRSA colonization of nares for ICU patients and additionally that nares colonization increased the risk for postoperative infections (Davis et al., 2004).

A study which conducted genetic analysis of surgical site infections found that among patients with *S. aureus* infections who were *S. aureus* nasal carriers, the wound infection isolates were identical to those of the nasal isolates and indicative of autoinfection (Ahmed et al., 1998). Similar findings were reported in a study that evaluated the relation between oro-nasal *S. aureus* and diabetic foot ulcers. Patients who had *S. aureus* present in both their oro-nasal area and ulcer sites were found to have identical strains of the bacteria in both locations (McManus et al., 2020). These findings highlight the significance of evaluating potential therapies for MRSA soft tissue infections against nasal isolates. To our knowledge, there are not many existing studies that have evaluated topical NO formulations for treating wounds infected with a nasal MRSA isolate.

Since 1991, the airflow of NO was found within the airways of humans and animals with studies showing that it accumulates during nasal cycle, speech, swallowing or even humming which may be subsequent with reducing the risk of sinusitis and other sinus infections when exhaling through the nose (Chatkin et al., 1999; Weitzberg and Lundberg, 2002). There are many functions for NO that have been incorporated into a diverse array of treatments beyond those for nasal and skin infections. Prevention and treatment of urinary tract infections have been evaluated by filling urinary catheter retention balloons with NO and ascorbic acid which subsequently prevented biofilm formation and decreased bacterial counts. Respiratory infections have been analyzed in animal and human trails with gaseous NO to improve airflow support and prevent or reduce infections, biofilm growth, pulmonary vascular resistance, and lung infiltrates for patients, especially with severe acute respiratory syndrome (SARS) (Bath et al., 2021).

As a free radical, NO cells can easily interact with reactive oxygen intermediates, such as hydrogen peroxide (H_2_O_2_) and superoxide (O_2_−) that target DNA and can make alternations for creating various antimicrobial molecular species (Doxey et al., 2020). Research has shown that NO can disseminate bacterial biofilms, and that the antibacterial mechanism of NO demonstrates limited bacterial elimination at low concentrations (usually below 1 μM) while a greater extent of bacterial eradication is observed at high concentrations (Rong et al., 2019). This aligns with our results which demonstrated that the higher concentration of NO (1.8%) exhibited more bacterial reductions on than the lower concentrations of NO on both assessment days (**Figure 4**). Treatment NVN 4428 (1.8%) + Hydrogel displayed significant differences of bacterial reductions over 2.58 Log CFU/mL (99.74%) on day 7 when compared to the other NO formulations. A higher concentration of NO than NVN 4428 (1.8%) may demonstrate substantial reductions and possible eradication if tested.

Our study shows a significant (p ≤ 0.05) decline of MRSA BAA1686 following treatment with each NO formulation, with a bacterial reduction greater than 96% observed between both assessment times. Similarly, an *in vivo* study evaluated antibacterial and anti-inflammatory abilities of titanium implants integrated with NO on MRSA biofilms achieved over 95.7% eradication after near-infrared light (NIR) irradiation with limited inflammatory cells and residual bacteria (Yu et al., 2023). The NVN 4428 (1.8%) + Hydrogel treated wounds displayed a bacterial difference of 3.31 ± 0.76 Log CFU/mL within 3 days, while the lower concentrations of NO exhibited reductions of about half that amount. While although the present study period was 7 days, all the topical NO formulations from day 4 to day 7 had significant (p ≤ 0.05) bacterial reductions over 1.45 Log CFU/mL within three days. A longer study duration may elucidate the potential for NO therapy to achieve pathogen eradication or more substantial reductions.

The results for this study demonstrate how effective topical NO formulation is against MRSA BAA1686. Although the treatment Mupirocin 2% was the best overall at extinguishing MRSA BAA1686, the NVN 4428 (1.8%) treatment performed just as well. Our study demonstrates that a topical NO-releasing formulation closely matches the antimicrobial efficiency of mupirocin which can lead to unlocking more antibiotic properties and development of new therapies. It may as well lead to being able to perform well against other microorganisms.

The present study evaluated NO only against Gram-positive MRSA but additional investigation into topical NO therapy against the Gram-negative *P. aeruginosa* (PA) or other common pathogenic microorganisms may provide additional insight into the range of antimicrobial efficacy. Innumerable reports from both *in vitro* and *in vivo* studies have publicized the efficacy of NO against numerous microorganisms, including fungi. (Stasko et al., 2018; Bath et al., 2021). Previously, NO-releasing polymeric fibers were demonstrated to have greater efficacy against PA than MRSA *in vitro* by observing viability of 46%-65% compared to 60%-86%, respectively (Wang et al., 2021). One study investigated a topical NO-releasing nanoparticle and demonstrated reductions of over 99% against PA, *S. aureus*, *E. coli*, MSSE and CA biofilms in culture, and approximately 90% of AB in infected murine wounds (Schairer et al., 2012). These various organisms could also be affected by the NVN 4428 formulations and may reveal some interesting results.

Most nasal infections are treated with proper nasal irrigations, sprays, steroids or oral antihistamines with sodium hyaluronate to help adults and children and prevent associated morbidities such as reduced productivity and psychological problems (De Corso et al., 2022). By Harnessing NO and being able to use and distribute it properly has led to multiple NO products successfully reducing microorganisms. This study shows the effectiveness of the NVN 4428 on a colonized intranasal specimen which may spread from the nose to other parts of the body and even other individuals.

Although effects on wound healing were not evaluated in the present study, many studies have also demonstrated the use of topical NO agents for wound healing and that this therapy has the potential to act as a suitable barrier from infections while accelerating wound healing (Man et al., 2022). NO has shown to regulate cytokines that initiate inflammation such as interleukins, monocytes, and neutrophils which lead to promoting acceleration to the wound healing process in general and also in burned wounds (Luo and Chen, 2005; Singer et al., 2018; Malone-Povolny et al., 2019). These findings ought to be further evaluated to not just eradicate microbes but also the potential for accelerating wound healing and its application within nares.

Over the past few years, the antibacterial properties of NO have drawn the interest of many researchers for a better understanding of its antibacterial mechanism (Jones et al., 2010). There are still multiple things to learn about the various functions of NO and how it collaborates with other materials to fulfill its utmost potential. As technology advances with nanotechnology and being developed into various wound dressings, an array of NO-releasing nanoparticle formulations has shown improvement on antibacterial and wound healing proficiencies (Lee et al., 2020).

## Conclusion

All treatment groups displayed a significant (p ≤ 0.05) decreasing trend of MRSA BAA1686 from one assessment day to the other. The NVN 4428 (1.8%) treatment reduced the most nasal strain biofilms of MRSA by outperforming the other NO formulations with lower concentrations on both assessment days. All treatment groups on Day 4 exhibited bacterial counts above the standard amount of infection (6 Log CFU/mL), while Mupirocin 2% alongside NO concentrations of 1.8% and 0.9% were the only treatments below the standard amount of infection on Day 7. A significant difference of over 2.58 Log CFU/mL in bacterial elimination was observed between the highest concentration of NO against the other NO formulations which supports its efficacy and ability to reduce nasal strains of MRSA biofilms. Every NO formulation may possess the ability to become more active over a certain timespan since all concentrations decreased the MRSA counts, however the most concentrated 1.8% expressed the greatest difference of reductional value between assessment days. Further investigations of the NO formulations and optimizations are needed to evaluate these results, to produce a more effective product with a greater potential of eliminating infections and biofilms.

## Data Availability

The original contributions presented in the study are included in the article/supplementary material. Further inquiries can be directed to the corresponding author.

## References

[B1] Abdel Azim S. Whiting C. Friedman A. J. (2024). Applications of nitric oxide-releasing nanomaterials in dermatology: Skin infections and wound healing. Nitric. Oxide 146, 10–18. doi: 10.1016/j.niox.2024.03.001 38458595

[B2] Ahmed A. O. A. van Belkum A. Fahal A. H. Elnor A. E. A. Abougroun E. S. A. M. VandenBergh M. F. Q.. (1998). Nasal carriage of Staphylococcus aureus and epidemiology of Surgical-site infections in a Sudanese university hospital. J. Clin. Microbiol. 36, 3614–3618. doi: 10.1128/jcm.36.12.3614-3618.1998 9817883 PMC105250

[B3] Almatroudi A. (2024). Investigating biofilms: advanced methods for comprehending microbial behavior and antibiotic resistance. Front. Biosci. (Landmark Ed). 29, 133. doi: 10.31083/j.fbl2904133 38682189

[B4] Augusto de Oliveira M. F. Agne D. B. Bastos L. S. S. Andrade de Oliveira L. M. Saintive S. Goudouris E. S.. (2024). Atopic dermatitis pediatric patients show high rates of nasal and intestinal colonization by methicillin-resistant Staphylococcus aureus and coagulase-negative staphylococci. BMC Microbiol. 24, 42. doi: 10.1186/s12866-023-03165-5 38287251 PMC10823624

[B5] Bath P. M. Coleman C. M. Gordon A. L. Lim W. S. Webb A. J. (2021). Nitric oxide for the prevention and treatment of viral, bacterial, protozoal and fungal infections. F1000Res 10, 536. doi: 10.12688/f1000research.51270.2 35685687 PMC9171293

[B6] Belenichev I. Popazova O. Bukhtiyarova N. Savchenko D. Oksenych V. Kamyshnyi O. (2024). Modulating nitric oxide: implications for cytotoxicity and cytoprotection. Antioxidants (Basel). 13, 504. doi: 10.3390/antiox13050504 38790609 PMC11118938

[B7] Bryan N. S. (2015). Nitric oxide enhancement strategies. Future Sci. OA 1, FSO48. doi: 10.4155/FSO.15.48 28031863 PMC5137939

[B8] Carbó E. Croasdell G. (2013). 53rd annual interscience conference on antimicrobial agents and chemotherapy (ICAAC), Denver, corolado, USA, September 10-13, 2013. Drugs Future. 38, 722. doi: 10.1358/dof.2013.038.10.2062686

[B9] Cartwright M. Enloe C. Stripling S. Maeda-Chubachi T. (2022). Pharmacokinetic profile, safety, and tolerability of topical berdazimer gel, 10.3% in patients with Molluscum contagiosum. J. Drugs Dermatol. 21, 1104. doi: 10.36849/JDD.6938 36219053

[B10] Chatkin J. M. Qian W. McClean P. A. Zamel N. Haight J. Silkoff P. (1999). Nitric oxide accumulation in the nonventilated nasal cavity. Arch. Otolaryngol Head Neck Surg. 125, 682–685. doi: 10.1001/archotol.125.6.682 10367927

[B11] Choi M. Hasan N. Cao J. Lee J. Hlaing S. P. Yoo J. W. (2020). Chitosan-based nitric oxide-releasing dressing for anti-biofilm and in *vivo* healing activities in MRSA biofilm-infected wounds. Int. J. Biol. Macromol. 142, 680–692. doi: 10.1016/j.ijbiomac.2019.10.009 31622708

[B12] Costa F. G. Mills K. B. Crosby H. A. Horswill A. R. (2024). The Staphylococcus aureus regulatory program in a human skin-like environment. mBio 15, e0045324. doi: 10.1128/mbio.00453-24 38546267 PMC11077960

[B13] Coye T. L. Foote C. Stasko P. Demarco B. Farley E. Kalia H. (2023). Predictive value of MRSA nares colonization in diabetic foot infections: A systematic review and bivariate random effects meta-analysis. J. Foot Ankle Surg. 62, 576–582. doi: 10.1053/j.jfas.2022.06.006 36922315

[B14] Cui T. Xu F. Wang J. Li W. Gao Y. Li X.. (2024). Polydopamine nanocarriers with cascade-activated nitric oxide release combined photothermal activity for the therapy of drug-resistant bacterial infections. ACS Infect. Dis 10, 2018–2031. doi: 10.1021/acsinfecdis.4c00021 38743862

[B15] Davis K. A. Stewart J. J. Crouch H. K. Florez C. E. Hospenthal D. R. (2004). Methicillin-resistant Staphylococcus aureus (MRSA) nares colonization at hospital admission and its effect on subsequent MRSA infection. Clin. Infect. Dis. 39, 776–782. doi: 10.1086/422997 15472807

[B16] Davis S. C. Harding A. Gil J. Parajon F. Valdes J. Solis M.. (2017). Effectiveness of a polyhexanide irrigation solution on methicillin-resistant Staphylococcus aureus biofilms in a porcine wound model. Int. Wound J. 14, 937–944. doi: 10.1111/iwj.12734 28266133 PMC7950165

[B17] Davis S. C. Li J. Gil J. Head C. Valdes J. Glinos G. D.. (2019). Preclinical evaluation of a novel silver gelling fiber dressing on Pseudomonas aeruginosa in a porcine wound infection model. Wound Repair Regen. 27, 360–365. doi: 10.1111/wrr.12718 30920083

[B18] Davis S. C. Li J. Gil J. Valdes J. Solis M. Treu R.. (2015). A closer examination of atraumatic dressings for optimal healing. Int. Wound J. 12, 510–516. doi: 10.1111/iwj.12144 24028503 PMC7950349

[B19] Davis S. C. Ricotti C. Cazzaniga A. Welsh E. Eaglstein W. H. Mertz P. M. (2008). Microscopic and physiologic evidence for biofilm-associated wound colonization in *vivo* . Wound Repair Regener. 16, 23–29. doi: 10.1111/j.1524-475X.2007.00303.x 18211576

[B20] De Corso E. Seccia V. Ottaviano G. Cantone E. Lucidi D. Settimi S. (2022). Clinical evidence of type 2 inflammation in non-allergic rhinitis with eosinophilia syndrome: a systematic review. Curr. Allergy Asthma Rep. 22, 29–42. doi: 10.1007/s11882-022-01027-0 35141844

[B21] Dexter F. Walker K. M. Brindeiro C. T. Loftus C. P. Banguid C. C. L. Loftus R. W. (2024). A threshold of 100 or more colony-forming units on the anesthesia machine predicts bacterial pathogen detection: a retrospective laboratory-based analysis. Can. J. Anaesth. 71, 600–610. doi: 10.1007/s12630-024-02707-3 38413516

[B22] Doxey R. Bao J. inventors; Novan Inc., assignee. (2020). Topical Compositions and Methods of using the Same. Patent number US 10,828,323 B2.

[B23] Ghaffari A. Miller C. C. McMullin B. Ghahary A. (2006). Potential application of gaseous nitric oxide as a topical antimicrobial agent. Nitric. Oxide 14, 21–29. doi: 10.1016/j.niox.2005.08.003 16188471

[B24] Gonzalez A. M. Townsend J. R. Pinzone A. G. Hoffman J. R. (2023). Supplementation with nitric oxide precursors for strength performance: A review of the current literature. Nutrients 15, 660. doi: 10.3390/nu15030660 36771366 PMC9921013

[B25] Goodwine J. Gil J. Doiron A. Valdes J. Solis M. Higa A.. (2019). Pyruvate-depleting conditions induce biofilm dispersion and enhance the efficacy of antibiotics in killing biofilms in vitro and in *vivo* . Sci. Rep. 9, 3763. doi: 10.1038/s41598-019-40378-z 30842579 PMC6403282

[B26] Gulati M. Thomas J. M. Ennis C. L. Hernday A. D. Rawat M. Nobile C. J. (2024). The bacillithiol pathway is required for biofilm formation in Staphylococcus aureus. Microb. Pathog. 191, 106657. doi: 10.1016/j.micpath.2024.106657 38649100

[B27] Gupta A. Dhingra A. (2018). Chronic rhinotillexomania leading to unilateral external nare stenosis. Cureus 10, e3172. doi: 10.7759/cureus.3172 30410823 PMC6207173

[B28] Jones M. L. Ganopolsky J. G. Labbé A. Wahl C. Prakash S. (2010). Antimicrobial properties of nitric oxide and its application in antimicrobial formulations and medical devices. Appl. Microbiol. Biotechnol. 88, 401–407. doi: 10.1007/s00253-010-2733-x 20680266

[B29] Lee J. Kwak D. Kim H. Kim J. Hlaing S. P. Hasan N.. (2020). Nitric oxide-releasing S-nitrosoglutathione-conjugated poly(Lactic-co-glycolic acid) nanoparticles for the treatment of MRSA-infected cutaneous wounds. Pharmaceutics 12, 618. doi: 10.3390/pharmaceutics12070618 32630779 PMC7407147

[B30] Luo J. D. Chen A. F. (2005). Nitric oxide: a newly discovered function on wound healing. Acta Pharmacol. Sin. 26, 259–264. doi: 10.1111/j.1745-7254.2005.00058.x 15715920

[B31] Malone-Povolny M. J. Maloney S. E. Schoenfisch M. H. (2019). Nitric oxide therapy for diabetic wound healing. Adv. Healthc Mater. 8, e1801210. doi: 10.1002/adhm.201801210 30645055 PMC6774257

[B32] Man M. Q. Wakefield J. S. Mauro T. M. Elias P. M. (2022). Role of nitric oxide in regulating epidermal permeability barrier function. Exp. Dermatol. 31, 290–298. doi: 10.1111/exd.14470 34665906 PMC8897205

[B33] McKinnell J. A. Bartsch S. M. Lee B. Y. Huang S. S. Miller L. G. (2015). Cost-benefit analysis from the hospital perspective of universal active screening followed by contact precautions for methicillin-resistant Staphylococcus aureus carriers. Infect. Control Hosp Epidemiol. 36, 2–13. doi: 10.1017/ice.2014.1 25627755 PMC4500653

[B34] McManus B. A. Daly B. Polyzois I. Wilson P. Brennan G. I. Fleming T. E.. (2020). Comparative microbiological and whole-genome analysis of staphylococcus aureus populations in the Oro-nasal cavities, skin and diabetic foot ulcers of patients with type 2 diabetes reveals a possible Oro-nasal reservoir for ulcer infection. Front. Microbiol. 11. doi: 10.3389/fmicb.2020.00748 PMC7212350 32425909

[B35] Mulcahy M. E. McLoughlin R. M. (2016). Host-bacterial crosstalk determines Staphylococcus aureus nasal colonization. Trends Microbiol. 24, 872–886. doi: 10.1016/j.tim.2016.06.012 27474529

[B36] NNIS System (2003). National Nosocomial Infections Surveillance (NNIS) System Report, data summary from January 1992 through June 2003, issued August 2003. Am. J. Infect. Control. 31, 481–498. doi: 10.1016/j.ajic.2003.09.002 14647111

[B37] Pastar I. Nusbaum A. G. Gil J. Patel S. B. Chen J. Valdes J.. (2013). Interactions of methicillin resistant Staphylococcus aureus USA300 and Pseudomonas aeruginosa in polymicrobial wound infection. PloS One 8, e56846. doi: 10.1371/journal.pone.0056846 23451098 PMC3579943

[B38] Rong F. Tang Y. Wang T. Feng T. Song J. Li P.. (2019). Nitric oxide-releasing polymeric materials for antimicrobial applications: A review. Antioxidants 8, 556. doi: 10.3390/antiox8110556 31731704 PMC6912614

[B39] Schairer D. O. Chouake J. S. Nosanchuk J. D. Friedman A. J. (2012). The potential of nitric oxide releasing therapies as antimicrobial agents. Virulence 3, 271–279. doi: 10.4161/viru.20328 22546899 PMC3442839

[B40] Sharma S. Pal S. Negi V. Juyal D. Sharma M. Prakash R. (2020). Staphylococcus aureus including MRSA nasal carriage among hospital exposed and unexposed medical students. J. Family Med. Prim Care 9, 4936–4941. doi: 10.4103/jfmpc.jfmpc_820_20 33209825 PMC7652201

[B41] Singer A. J. Choi Y. Rashel M. Toussaint J. McClain S. A. (2018). The effects of topical nitric oxide on healing of partial thickness porcine burns. Burns J. Int. Soc. Burn Injuries 44, 423–428. doi: 10.1016/j.burns.2017.07.017 28869060

[B42] Stasko N. McHale K. Hollenbach S. J. Martin M. Doxey R. (2018). Nitric oxide-releasing macromolecule exhibits broad-spectrum antifungal activity and utility as a topical treatment for superficial fungal infections. Antimicrob. Agents Chemother. 62, e01026–e01017. doi: 10.1128/AAC.01026-17 29760128 PMC6021618

[B43] Sullivan T. P. Eaglstein W. H. Davis S. C. Mertz P. (2001). The pig as a model for human wound healing. Wound Repair Regen. 9, 66–76. doi: 10.1046/j.1524-475x.2001.00066.x 11350644

[B44] Troeman D. P. R. Kluytmans J. A. J. W. (2023). From Nares to Wound: Exploring the mechanisms for Staphylococcal surgical site infections, implications for infection prevention. Antimicrob. Steward Healthc Epidemiol. 3, e130. doi: 10.1017/ash.2023.197 37592968 PMC10428146

[B45] Wang D. C. Clark J. R. Lee R. Nelson A. H. Maresso A. W. Acharya G.. (2021). Development of antimicrobial nitric oxide-releasing fibers. Pharmaceutics 13, 1445. doi: 10.3390/pharmaceutics13091445 34575520 PMC8468281

[B46] Weitzberg E. Lundberg J. O. (2002). Humming greatly increases nasal nitric oxide. Am. J. Respir. Crit. Care Med. 166, 144–145. doi: 10.1164/rccm.200202-138BC 12119224

[B47] Wolde W. Mitiku H. Sarkar R. Shume T. (2023). Nasal carriage rate of staphylococcus aureus, its associated factors, and antimicrobial susceptibility pattern among health care workers in public hospitals, Harar, Eastern Ethiopia. Infect. Drug Resist. 16, 3477–3486. doi: 10.2147/IDR.S396570 37287547 PMC10243340

[B48] Wos-Oxley M. L. Chaves-Moreno D. Jáuregui R. Oxley A. P. Kaspar U. Plumeier I.. (2016). Exploring the bacterial assemblages along the human nasal passage. Environ. Microbiol. 18, 2259–2271. doi: 10.1111/1462-2920.13378 27207744

[B49] Yang Z. Qiu B. Cheng D. Zhao N. Liu Y. Li M.. (2022). Virulent Staphylococcus aureus colonizes pediatric nares by resisting killing of human antimicrobial peptides. Int. J. Med. Microbiol. 312, 151550. doi: 10.1016/j.ijmm.2022.151550 35091347

[B50] Yu Y. L. Wu J. J. Lin C. C. Qin X. Tay F. R. Miao L.. (2023). Elimination of methicillin-resistant Staphylococcus aureus biofilms on titanium implants via photothermally-triggered nitric oxide and immunotherapy for enhanced osseointegration. Mil Med. Res. 10, 21. doi: 10.1186/s40779-023-00454-y 37143145 PMC10158155

